# External-Force-Offset Effects of ECM Coating Layers on hMSCs Subjected to External Physical Force

**DOI:** 10.34133/bmr.0265

**Published:** 2025-10-03

**Authors:** Cho Young Park, Kyoung Choi, Young-Jin Kim, Seok Chung, Jun Shik Choi, Sang Jun Park, Chun-Ho Kim

**Affiliations:** ^1^ Laboratory of Tissue Engineering, Korea Institute of Radiological and Medical Sciences, Seoul 01812, Republic of Korea.; ^2^ Program in Biomicro System Technology, Korea University, Seoul 02841, Republic of Korea.; ^3^ Department of Advanced Materials and Chemical Engineering, Daegu Catholic University, Gyeongsan, Gyeongbuk, Republic of Korea.

## Abstract

Mesenchymal stem cells (MSCs) used for cell-delivery-based therapy also undergo considerable external stresses upon entering the recipient site in the body. Here, we sought to develop a cell-protective barrier on the MSC surface that protects against stress-induced damage from physical external stresses. The barrier was fabricated from gelatin and hyaluronic acid (HyA) using a layer-by-layer (LbL) technique. In addition to assessing the stability and biological properties of extracellular matrix (ECM)-coated human bone marrow-derived MSCs (hMSCs) produced using the LbL, we also evaluated the cell-protective effects of this coating against 2 external stresses: low-attachment conditions and mechanical force induced by injection. Cell biological and morphological surface changes accompanying cell surface coating were analyzed using fluorescence-activated cell sorting and scanning electron microscopy. Viability and cell cycle characteristics were not substantially different between bare hMSCs and ECM-coated hMSCs with different numbers of layers after 7 days in culture. Stemness was also maintained, as reflected in >97.3% expression of positive markers and <0.5% expression of negative markers in 6-layered ECM-coated hMSCs, termed ECM-hMSCs. ECM-hMSCs showed 62.1% decrease in cell damage and 50.6% increase in DNA content after 3 days under low-attachment conditions. In addition, ECM-hMSCs injected at 100 and 200 kPa showed 27.2% and 41.8% higher viability, with damaged cells decreased by 54.9% and 45.6%, respectively, compared to bare hMSCs. These results show that LbL coating of hMSCs with gelatin and HyA does not impair the function of hMSCs and can physically protect cells from low-attachment conditions and the mechanical force associated with injection.

## Introduction

Stem cells are a promising candidate for cell therapy, regenerative medicine, and tissue repair applications, reflecting their self-renewal, multilineage-differentiation, and immunomodulatory properties [1,2]. Mesenchymal stem cells (MSCs), in particular, have been applied clinically for intervertebral disc and inflammatory diseases [3]. The therapeutic efficacy of stem cells has been limited by the vulnerability of cells to external factors, such as physical stress and microenvironmental changes, that negatively impact cell viability [4]. Following arrival at their recipient site in the body, MSCs can be exposed to various external stressors, including mechanical forces (e.g., shear stress from injection through a small-bore needle) and microenvironmental challenges, such as hypoxia, environmental proteases, and inflammation [5,6]. These factors can play significant roles in cellular growth and differentiation. Some studies have shown that moderate stimuli, such as periodic stretching, electrical conductivity, low-dose irradiation, and cyclic strain, can enhance cellular viability and facilitate cellular biological functions in vitro [7–10]. All types of excessive stress, however, have severe adverse effects on the death of cells or the development of diseases. Several studies have reported on cellular behaviors induced by hydraulic pressure or flow-induced shear stress, particularly in relation to cellular senescence and responses to an inflammatory environment [11–13].

MSCs exposed to these external stresses could experience damage to cell membranes or DNA, as well as leakage of cytosol components leading to cell necrosis and apoptosis [8,14]. For example, injection of cells into the human body through a syringe needle exposes cells to shear stress due to linear shear flow and stretching forces due to extensional flow [6]. These mechanical factors lead to a reduction in cell viability as a result of plasma membrane damage [14]. In addition, MSCs in culture must be detached from their surrounding matrix or cell culture substrate before injecting [15]. During the isolation process, loss of signaling pathways linked to the extracellular matrix (ECM) can be disrupted, leading to the death of MSCs through a process called anoikis, a particular type of cell death that occurs as a result of detachment from the ECM [14]. Anoikis can also undermine the efficacy of MSCs in tissue engineering and regenerative medicine applications, including osteoarthritis, myocardial infarction, and ischemic stroke [16–18]. A previous report showed that the post-transplantation viability was as low as 1% to 32% on cardiomyocytes, severely reducing the therapeutic efficacy of cells [19]. In preclinical studies on MSC transplantation, cells have been reported to be detected at 2.3% to 5.1% in cartilage defects, 0.01% to 0.3% in the distal femur and proximal tibia, and 6.1% to 8.4% at the site of spinal cord injury [20–22]. However, studies on protecting MSCs from physical external stresses have not been reported.

In this context, we used a layer-by-layer (LbL) self-assembly technique to protect MSCs from various external stresses. The LbL technique is widely favored for its ease of use and simplicity, which facilitates its broad applicability in various biomedical and therapeutic fields [23–25]. It is also used to fabricate nanofilms on cell surfaces, depositing biocompatible material that mimics ECM microenvironments on a single-cell scale [23]. The coating on cells not only allows the continued exchange of oxygen and nutrients through membranes but also offers biocompatibility without loss of signaling pathways between the cell and matrix in culture [26]. Anchorage-dependent cells coated with cell-friendly biomaterials are expected to be protected from various external stresses, with previous studies reporting that coating of MSCs with gelatin/heparin inhibits pyroptosis, and that coating of mammalian cells with tannic acid/ferric ions protects against ultraviolet irradiation [27,28].

Gelatin, a representative cell-friendly biomaterial, is derived from collagen, which has a linear structure that mainly contains glycine, proline, and hydroxyproline [29]. Because of its lower cost and enhanced biodegradability compared with collagen, gelatin is widely used in hydrogels, nanofibers, and as a cell transplantation carrier [30,31]. The 3-peptide sequence, arginine–glycine–aspartic acid (RGD), in gelatin is recognized as a significant cell-binding motif by integrin (α5β1) on the cell surface [31]. Gelatin, together with α5β1 integrin, supports cell attachment, migration, and proliferation properties of cells [29]. Hyaluronic acid (HyA), another cell-friendly biomaterial, is a nonsulfated glycosaminoglycan and a major component of the ECM. HyA is known to interact with the cell surface receptor CD44, which is involved in interactions between cells and the ECM [32]. CD44 interacts with carboxylic acid in HyA and has roles in cell–cell interactions, cell–ECM adhesion, and cell migration [33,34]. These biological properties of ECMs can reduce anoikis caused by detachment of anchorage-dependent cells from the ECM. LbL coating of the surface of MSCs with gelatin and HyA provides each individual MSC with its own ECM-mimetic microenvironment.

**Fig. 1.**
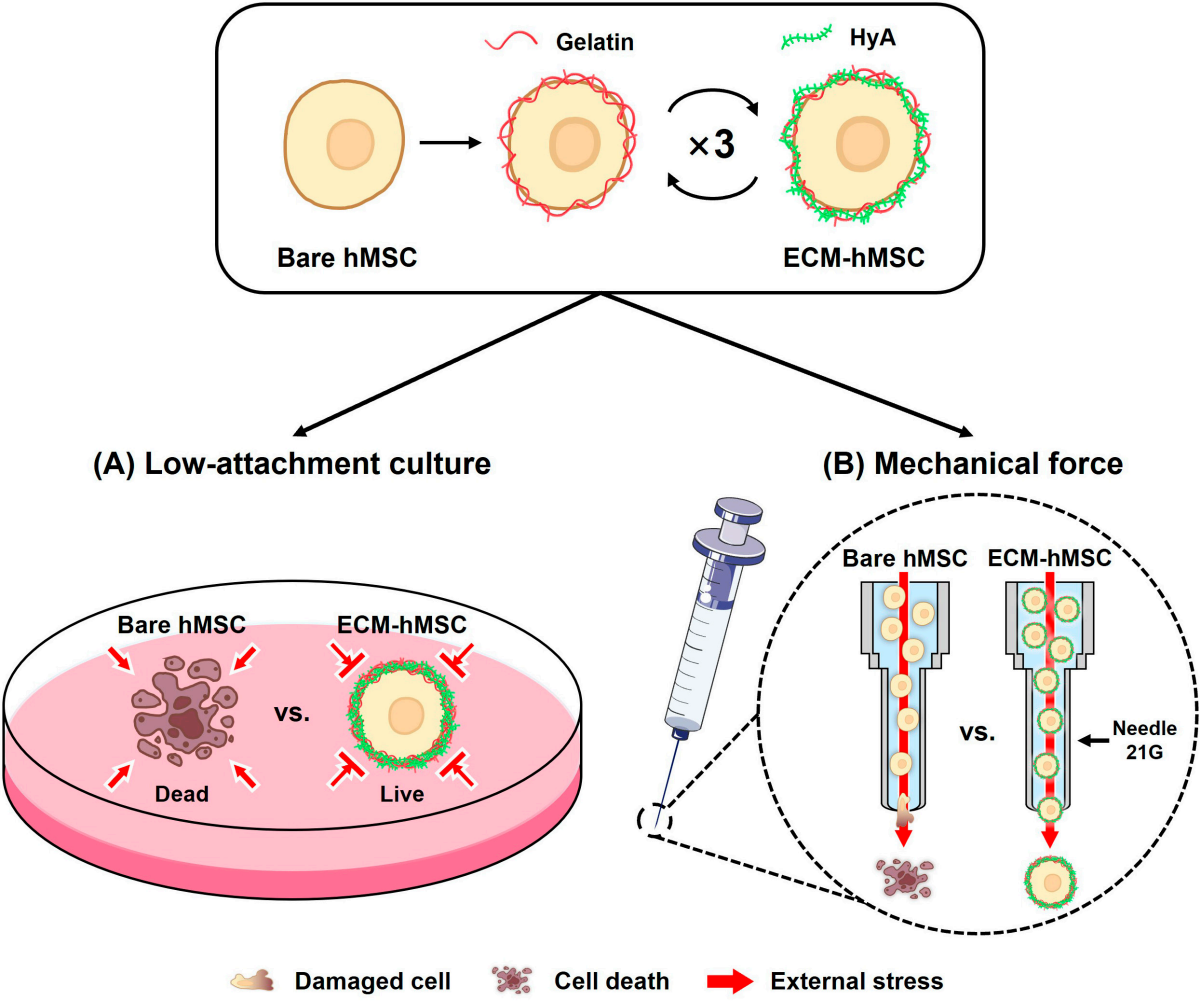
Schematic illustration of the ECM coating technique to enhance protective effect under various external stresses. ECM coating protects hMSCs under 2 different stress paradigms: (A) low-attachment culture conditions inducing anoikis and (B) mechanical forces simulated by needle injection using pneumatic pressure.

The goal of this study was to fabricate an ECM coating that protects human bone marrow-derived MSCs (hMSCs) from physical external stresses, as shown in Fig. 1. Individual hMSCs were coated with gelatin and HyA using an LbL self-assembly technique. The resulting ECM coating was evaluated by monitoring fluorescence changes on the coated cell surface, the diameter of ECM-coated hMSCs (ECM-hMSCs), and changes in cell morphology. The effects of ECM coating on the activity and stability of hMSCs were evaluated by assessing cell viability, cell proliferation, cell cycle progression, and maintenance of stemness. Finally, the ability of LbL-coated ECM to protect hMSCs from physical external stress was evaluated under low-attachment conditions and in response to the mechanical force imposed by injection.

## Materials and Methods

### Cell study

hMSCs (passages 4 to 6), seeded at a concentration of 2.5 × 10^5^ cells/dish in a 100-mm^2^ culture dish, were cultured in Mesenchymal Stem Cell Growth Medium (Lonza, Houston, TX, USA) in a humidified CO_2_ incubator. The media was changed every 2 to 3 days until cells reached 70% to 80% confluence. Cells were harvested for each experiment by first washing 2 times with Dulbecco’s phosphate-buffered saline (DPBS) (Lot. LB001250102, WelGENE, Daegu, Korea) and detached by incubating with a 0.05% trypsin/ethylene diamine tetraacetic acid (EDTA) (Lot. LS15230801, WelGENE, Daegu, Korea) solution.

### LbL ECM coating of hMSCs

Gelatin (Bloom 220-310, type A, MP Biomedicals, Irvine, CA, USA) was dissolved in DPBS at a concentration of 0.2% (w/w) at 37 °C for 4 h. HyA (10 kDa, Lifecore Biomedical, Chaska, USA) was dissolved in DPBS at a concentration of 0.1% (w/w) at 4 °C overnight. Each well of a 6-well plate (Lot. AB4H26A3506, SPL Insert Hanging, SPL Life Sciences, Gyeongsan, Korea) was filled with 2.5 ml of a 0.2% (w/w) gelatin/0.1% (w/w) HyA solution. A suspension of hMSCs (1 × 10^7^ cells in 500 μl of gelatin solution) was then added to a 6-well insert with a 3-μm pore membrane. hMSCs suspended in gelatin solution were incubated for 5 min on a horizontal orbital shaker (DSR-2800P, Nasco Korea Corp., Seoul, Korea) inside a CO_2_ incubator, then transferred to another well containing DPBS for removal of unbound excess gelatin; this constituted the first coating layer (Layer 1). For the next (HyA) layer coating, gelatin-coated hMSCs were transferred to another well containing a 0.1% (w/w) HyA solution. They were again incubated on a horizontal orbital shaker inside a CO_2_ incubator for 5 min and then transferred to another well containing DPBS for the removal of unbound excess HyA, forming Layer 2. This coating and washing process was repeated until all remaining coating layers (Layers 3 to 8) were deposited.

### Synthesis of fluorescence-labeled gelatin and HyA

Fluorescence-labeled gelatin-RBITC (rhodamine B isothiocyanate, Lot. SHBR3111, Sigma-Aldrich, USA) was synthesized according to previous methods [35]. Briefly, an RBITC solution was prepared by dissolving 5.36 mg of RBITC in 1 ml of dimethyl sulfoxide (DMSO, Lot. SHBR8851, Sigma-Aldrich, St. Louis, MO, USA). This solution was then slowly added dropwise at 37 °C into a stirred gelatin solution (prepared by dissolving 100 mg of gelatin in 20 ml of 0.2 M sodium bicarbonate [Lot. 38H0031, Sigma-Aldrich, St. Louis, MO, USA] buffer) wrapped in aluminum foil. After allowing the reaction mixture to stand overnight, unreacted reagents were removed by dialyzing the mixture against deionized water (DIW) for 5 days. Fluorescence-labeled gelatin-RBITC, obtained by freeze-drying for 3 days, was stored at −20 °C in the dark until use.

Fluorescence-labeled HyA-FITC (fluorescein isothiocyanate, Lot. 0000443588, Sigma-Aldrich, St. Louis, MO, USA) was synthesized according to previous methods [36]. Briefly, an FITC solution, prepared by dissolving 40 mg of FITC in 1 ml of DMSO, was slowly added (dropwise) into a stirred HyA solution (prepared by dissolving 50 mg of HyA in 9 ml of 0.1 M sodium bicarbonate buffer) wrapped in aluminum foil, and incubated at room temperature with continued stirring for 2 days. After centrifugation (2,000 rpm, 4 °C, 3 min), the precipitate was washed with cold absolute ethanol (EtOH), followed by centrifugation. The precipitate was dissolved in DIW and dialyzed against DIW for 2 days to remove unreacted reagents. Fluorescence-labeled HyA-FITC, obtained by freeze-drying for 3 days, was stored at −20 °C in the dark until use.

### Fluorescence-activated cell sorting was used to analyze ECM coating on hMSCs

Changes in cell characteristics upon the addition of coating layers were investigated by measuring fluorescence intensity by flow cytometry using fluorescence-activated cell sorting (FACS; CytoFLEX, Beckman Coulter, USA). The mean fluorescence intensity (MFI) of each gelatin-RBITC and/or HyA-FITC coating layer of ECM-coated hMSCs was measured at wavelengths of 561 and 488 nm, respectively. A total of 10,000 cells were measured in each group.

### Confocal laser scanning microscopy was used to analyze hMSC morphology after ECM coating

Surface morphological changes in bare hMSCs and ECM-hMSCs were visualized by confocal laser scanning microscopy (CLSM; LSM 880, Carl Zeiss, Oberkochen, Germany) detection of gelatin-RBITC and HyA-FITC deposited on the cell surface. To this end, cells were first fixed with 4% paraformaldehyde (PFA, Lot. P3123Y22M, Biosesang, Seongnam, Korea) then washed 3 times with 1× phosphate-buffered saline (PBS, Lot. LB204230202, WelGENE, Daegu, Korea). Thereafter, 200 μl of coated cell suspension (2 × 10^5^ cells in 1× PBS) was added to a confocal dish (Lot. BB0K02A100350, SPL Life Sciences, Gyeongsan, Korea), and absorbance was measured at wavelengths of 543 and 488 nm for gelatin-RBITC (red) and HyA-FITC (green), respectively.

### Scanning electron microscopy was used to analyze hMSC morphology after ECM coating

The surface morphology of bare hMSCs and ECM-coated hMSCs was further visualized by scanning electron microscopy (SEM; CX-200TM, COXEM, Daejeon, Korea). After ECM coating, cells were washed 3 times with DPBS and fixed with 4% PFA. Fixed cells were then washed 3 times with 1× PBS and dehydrated using a graded EtOH series (30% to 100%). Cell morphology was preserved by incubating fixed, dehydrated cells with hexamethyldisilazane (Lot. BCBN1583V, Sigma-Aldrich, St. Louis, MO, USA) at room temperature and then coated with gold using an ion sputter coater (SPT-20, COXEM, Daejeon, Korea). The surfaces of cells were imaged by SEM at 20 kV.

### Annexin V/PI staining was used to analyze hMSC viability by coating layers

Possible changes in cell viability induced by ECM coating were evaluated by annexin V/propidium iodide (PI) staining using an Annexin V-FITC apoptosis detection kit I (Lot. 556547, BD Pharmingen, Heidelberg, Germany), according to the manufacturer’s protocol. Briefly, cultured bare hMSCs or ECM-coated hMSCs were harvested, washed 2 times with DPBS, and dispersed in 100 μl of 1× annexin V binding buffer. Each cell type was stained by incubating with 2.5 μl of annexin V and 5 μl of PI at room temperature in the dark for 15 min and assessed by FACS analysis.

### Live/dead assay was used to analyze hMSC viability by coating layers

After different culture time points, cell viability was assessed by staining cells using a LIVE/DEAD Viability/Cytotoxicity kit (Lot. 2776013, Invitrogen, Carlsbad, CA, USA), according to the manufacturer’s protocol. Briefly, after removal of culture medium, wells were washed 3 times with DPBS, then stained with 1 μM calcein AM in 1.5 ml of media and 2 μM ethidium homodimer (Ethd-1) in media at 37 °C for 45 min. Stained cells were imaged by CLSM.

### MTT assay was used to analyze hMSC proliferation by coating layers

Effect of ECM coating on hMSC proliferation was evaluated using an EZ-Cytox kit (Lot. DLS2404, DoGenBio, Seoul, Korea). Bare hMSCs or ECM-coated hMSCs at a concentration of 1 × 10^4^ cells/well were seeded in 24-well cell culture plates and cultured in a CO_2_ incubator. After the desired time, cells were transferred to a 10% Ez-Cytox solution in media and incubated at 37 °C for 2 h. Absorbance was then measured in a 96-well UV plate at 450 nm using a UV–Vis spectrophotometer (SpectraMax M2e, Molecular Device Co., Ltd., San Jose, CA, USA).

### Cell cycle of bare hMSCs and ECM-hMSCs

Changes in the cell cycle after ECM coating were evaluated by first culturing bare hMSCs and ECM-hMSCs in 6-well plates at a concentration of 5 × 10^4^ cells/well for 7 days. Cells were then trypsinized, washed twice with DPBS, and fixed by incubating with cold 70% EtOH for 1 h. After washing 3 times with 1× PBS, fixed cells were incubated with 5 μl of PI, 0.5 μl of Triton X-100 (Lot. 121296, USB Corporation, Cleveland OH, USA), and 50 μl of ribonuclease A solution (100 μg/ml, Lot. 20917, Lucigen, USA) for 30 min. Changes in the cell cycle in each group were evaluated by FACS analysis.

### Stemness of bare hMSCs and ECM-hMSCs

The effects of ECM coating on the maintenance of ECM-hMSC stemness were evaluated using a panel of stemness markers of MSCs, according to the guidelines of The International Society for Cellular Therapy (ISCT) [37]. FITC-conjugated antibodies against CD29 (integrin beta 1, Lot. 448461, MOUSE ANTI HUMAN CD29: FITC Bio-Rad Formerly AbD Serotec), the HyA receptor CD44 (Lot. 160056, Mouse anti Human CD44: FITC Bio-Rad Formerly AbD Serotec), the ecto-5′-nucleotidase CD73(Lot. 149933 Mouse anti Human CD73: FITC Bio-Rad Formerly AbD Serotec), CD90 (Thy-1, Lot. 153037, Mouse anti Human CD90: FITC Bio-Rad Formerly AbD Serotec), and CD105 (endoglin, Lot. 158807, Mouse anti Human CD105: FITC Bio-Rad Formerly AbD Serotec) were used as positive markers of stemness, and FITC-conjugated antibodies against the hematopoietic progenitor CD34 (Lot. 2518336, CD34 Monoclonal Antibody [RAM34], FITC, eBioscience Invitrogen) and leukocyte common antigen CD45 (Lot. 2452789, CD45 Monoclonal Antibody [30-F11] FITC, eBioscience Invitrogen) were used as negative stemness markers. All antibodies were used at a dilution of 1:100. hMSCs at a concentration of 1 × 10^6^ cells/ml were rinsed with 5% bovine serum albumin (BSA; Lot. SLBN6510V, Sigma-Aldrich, St. Louis, MO, USA), in 1× PBS) at room temperature for 15 min. Afterward, antibody (0.1 μg/ml) was added and cells were incubated at 4 °C in the dark for 40 min. The cells were washed again with a 5% BSA solution and centrifuged (1,500 rpm, 4 °C, 3 min). The resulting pellets were resuspended in 500 μl of 1× PBS and evaluated by FACS analysis.

### Lactate dehydrogenase cytotoxicity assay was used to analyze cell damage under external stress conditions

Lactate dehydrogenase (LDH) cytotoxicity assays were performed according to the manufacturer’s protocol provided with the EZ-LDH kit (Lot. DLS2409, DoGenBio, Seoul, Korea). Briefly, cells were plated at a concentration of 7.5 × 10^3^ cells/well in a 96-well plate. After the desired time, 10 μl of lysis solution was added into each well of high control and volume control groups and plates were incubated at room temperature for 5 min. After lysis, the medium was centrifuged at 600×*g* for 10 min, and 10 μl of supernatant was transferred to a new 96-well plate. LDH Reaction Mixture (100 μl) was added into each well of the new plate and the plate was incubated at room temperature for 30 min in the dark. Released LDH was measured at 450 nm using a UV–Vis spectrophotometer.

### DNA assay was used to analyze cell viability under low-attachment conditions

The protective effect of ECM coating on hMSCs under low-attachment conditions was evaluated by first seeding cells at a concentration of 3 × 10^4^ cells/well in 24-well low-attachment plates (Lot. BB5D14A39724, SPL3D cell floater plate, SPL Life Sciences, Gyeongsan, Korea) and culturing for 3 days. DNA content was then measured using a Quanti-iT PicoGreen dsDNA Assay (Lot. 2816115, Invitrogen, Eugene, OR, USA), according to the manufacturer’s protocol. Briefly, after collecting cells in 24-well low-attachment plates, 300 μl of radioimmunoprecipitation assay buffer was added and cells were sonicated (30 s, power 50%, cycle 5) and centrifuged (1,000 rpm, 4 °C, 5 min). Thereafter, 20 μl of the resulting supernatant was added together with 80 μl of 1× Tris-EDTA buffer and 100 μl of PicoGreen reagent in a 96-well UV plate, and the plate was incubated at room temperature in the dark for 5 min. Fluorescence was measured at excitation and emission wavelengths of 480 and 520 nm, respectively, using a UV–Vis spectrophotometer.

### Flow cytometry with annexin V/PI dual staining was used to analyze cell viability under mechanical force

Suspensions of bare hMSCs or ECM-hMSCs at a concentration of 1 × 10^6^ cells/ml were loaded into a syringe and extruded through a 21-gauge (G) needle (Kovax-needle, Korea Vaccine Co., Ltd., Korea) under different pneumatic pressures (50, 100, 200, and 400 kPa) using a dispenser (ML-5000XII-CTR, Musashi Engineering, Japan). After passing through the needle, the extruded cells were immediately collected and dispersed in 100 μl of 1× annexin V binding buffer and stained with 2.5 μl of annexin V and 5 μl of PI at room temperature for 15 min in the dark. The viability of stained cells was assessed by FACS analysis.

### Statistical analysis

All experiments were separately repeated in at least triplicate, and data are presented as means ± SDs. Statistical analyses were performed using Student’s *t* test. A *P* value < 0.5 was considered significant; individual *P* values (*P < 0.05, **P < 0.01, and ***P < 0.001) are indicated in figure legends.

## Results

### Coating of hMSCs with ECM components

**Fig. 2.**
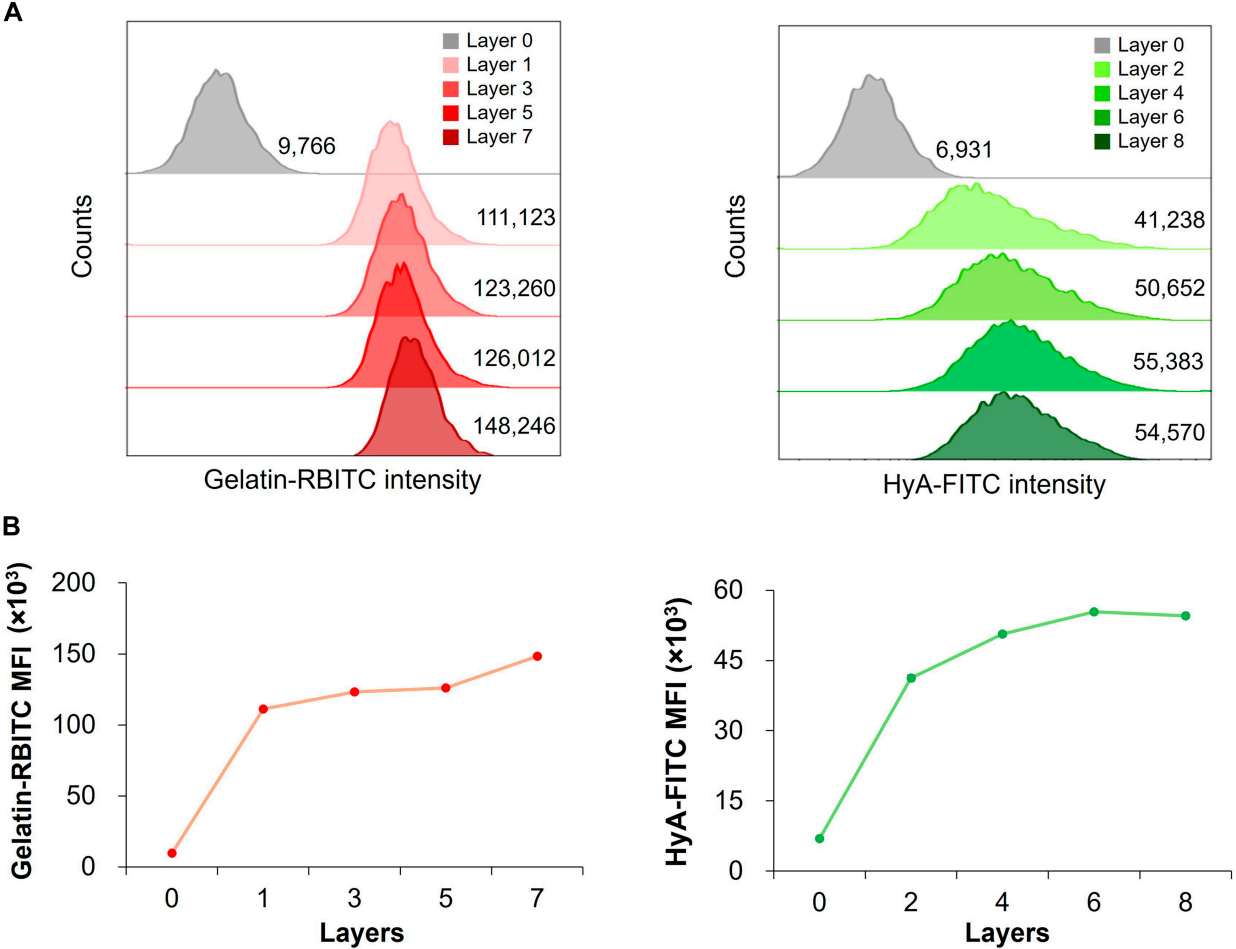
ECM coating using the layer-by-layer (LbL) technique was deposited on the cell surface. (A) Gelatin-RBITC/HyA-FITC-coated hMSCs via FACS analysis were shifted to the positive region (Gelatin-RBITC in red fluorescence and HyA–FITC in green fluorescence). (B) Mean fluorescence intensity (MFI) of the ECM coating layer on hMSC was changed with deposition of increasing numbers of layers based on FACS analysis (*n* = 4).

To confirm the coating of hMSCs with ECM components using the LbL technique, we used fluorescently tagged gelatin and HyA in the coating process. RBITC was used as a fluorescent reagent for gelatin, and FITC was used for HyA. A FACS analysis (Fig. 2A) showed that peaks of Gelatin-RBITC intensity (red) and HyA-FITC intensity (green) in all groups of ECM-coated hMSCs were shifted to more positive regions compared with peaks of bare hMSCs (gray). The MFI, calculated from Fig. 2A, of Gelatin-RBITC increased by 11.4 ± 0.1-, 12.6 ± 0.2-, 12.9 ± 0.1-, and 15.2 ± 0.1-fold with the application of Layers 1, 3, 5, and 7, respectively, compared with that of bare hMSCs (Layer 0), as shown in Fig. 2B. The MFI of HyA-FITC, in turn, increased by 6.0 ± 0.1-, 7.3 ± 0.1-, 8.0 ± 0.1-, and 7.9 ± 0.1-fold with the application of Layers 2, 4, 6, and 8, respectively, compared with that of bare hMSCs (Layer 0). In both Gelatin-RBITC and HyA-FITC groups, the MFI increased substantially after coating with only a single layer (Layer 1 and Layer 2 in Fig. 2). MFI continued to increase with increased numbers of coating layers until the final Layer 8 coating with HyA. For HyA-coated groups, the MFI gradually increased with increases in the number of coating layers up to Layer 6. However, Layer 8 showed a similar or a lower index without any further increase compared to Layer 6 (Fig. 2).

### Effects of ECM coating on cell viability and proliferation

**Fig. 3.**
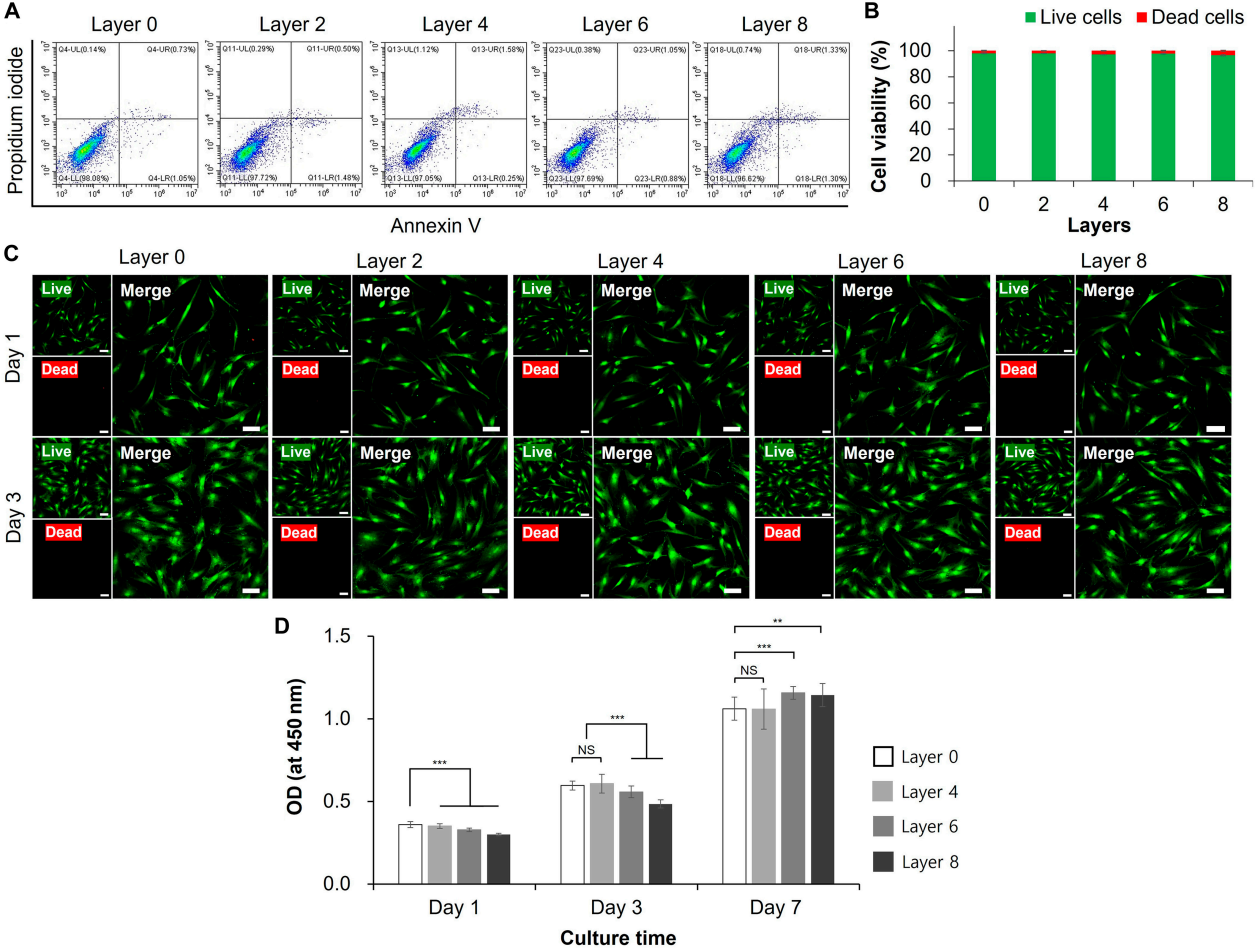
The viability and proliferation of bare hMSCs and ECM-coated hMSCs to establish optimal conditions for ECM coating. (A) The annexin V/PI images revealed the cell distribution at 5 min coating per layer. (B) Summarized bar graph illustrating the percentage of cell viability from (A) (*n* = 4). (C) The live/dead images of hMSCs at each coating layer on days 1 and 3 of cultures using calcein-AM/ethidium homodimer (Ethd-1) staining. (scale bars:100 μm). (D) Proliferation of hMSCs at each coating layer on days 1, 3, and 7 of cultures using the MTT assay (*n* = 4, **P < 0.01 and ***P < 0.001; NS, not significant).

To establish optimal conditions for ECM coating, we evaluated the viability and proliferation of hMSCs as a function of the number of coating layers. Changes in cell viability were evaluated based on a FACS analysis of the ratio of annexin V-stained to PI-stained cells (Fig. 3A and Fig. S1). In all groups, the viability of coated cells for 10-min coating per layer was slightly reduced compared to those of 5-min coating per layer (Fig. S1). A quantitative analysis of flow cytometry results of 5-min coating per layer (Fig. 3B) showed that the viability of bare hMSCs was 98.1% ± 0.3%, a value that was not significantly changed by coating hMSCs with 2 (98.0% ± 0.2%), 4 (97.1% ± 0.2%), 6 (97.8% ± 0.3%), or 8 (96.6% ± 0.5%) ECM layers. Further increasing the number of repeated coatings slightly, but insignificantly, decreased cell viability. These findings were also confirmed by live/dead cell assays performed at different culture times (Fig. 3C), which showed that most cells appeared viable, with so few dead cells that they were nearly undetectable. Increasing the number of coatings did not lead to a further decrease in cell viability, measured after 3 days in culture. Collectively, these results indicate no significant difference in viability between bare hMSCs and ECM-coated hMSCs with different numbers of layers. We next assessed the proliferation of ECM-coated hMSCs according to the number of coatings at various times during a 7-day culture period using 3-(4,5-dimethylthiazol-2-yl)-2,5-diphenyltetrazolium bromide (MTT) assays. As shown in Fig. 3D, the proliferation of ECM-coated hMSCs, determined from optical density (OD) measurements, increased for each ECM layer group over time. On day 1, OD values decreased slightly with increasing number of coating layers. At the 3-day mark, the proliferation of 6-layer and 8-layer hMSCs was lower than that of 0-layer and 4-layer hMSCs. However, by day 7, the OD values for 6-layer and 8-layer hMSCs had slightly exceeded that of 0-layer and 4-layer hMSCs, which exhibited proliferation levels similar to each other. Based on these results, we used 6-layer ECM-coated hMSCs with a final HyA coating layer, termed ECM-hMSCs, in subsequent experiments.

### Effects of ECM coating on cell morphology

**Fig. 4.**
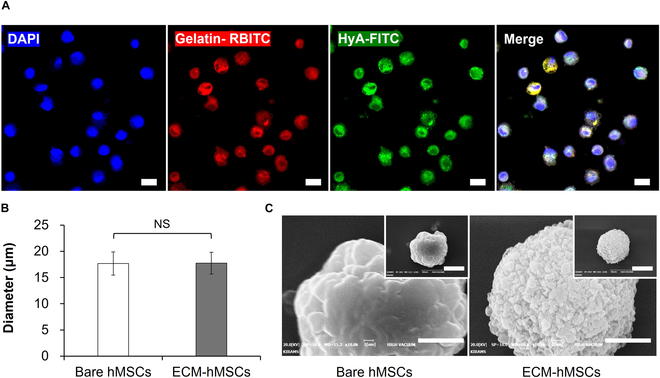
Surface morphological changes in bare hMSCs and ECM-hMSCs. (A) Confocal laser scanning microscopy (CLSM) images of Gelatin-RBITC (red)/HyA-FITC (green)-coated hMSCs. The nuclei were stained with 4′,6-diamidino-2-phenylindole (DAPI) in blue (magnification: ×400, scale bar: 20 μm). (B) The cell diameter of bare hMSCs and ECM-hMSCs was calculated using ImageJ based on the images of CLSM (*n* = 50). (C) Scanning electron microscope (SEM) image depicting the cell surface morphology between the bare hMSCs and ECM-hMSCs independent experiments (inner image—magnification: ×5.0 k, scale bar: 10 μm; outer image—magnification: ×10.0 k, scale bar: 5 μm).

The morphologies of bare hMSCs and ECM-hMSCs were assessed using CLSM and SEM. The overall morphologies of the ECM-hMSCs with a final HyA coating layer are shown in Fig. 4. Fluorescence images of ECM-hMSCs (Fig. 4A) show a close overlap of gelatin (red) and HyA (green) fluorescence in ECM layers with 4′,6-diamidino-2-phenylindole (DAPI) staining of cell nuclei (blue). ECM-hMSCs appeared as single cells with homogeneous coatings, indicating that the presence of ECM coating layers did not cause cell aggregation. Fluorescence images obtained by CLSM further showed no significant difference in the diameter of bare hMSCs (17.67 ± 2.21 μm) or ECM-hMSCs (17.74 ± 2.07 μm) (Fig. 4B). In addition, SEM images showed differences in the surface morphology of cells before and after ECM coating (Fig. 4C), revealing that ECM-hMSCs had a rougher surface than bare hMSCs.

### Effects of ECM coating on the cell cycle and the maintenance of hMSC stemness

**Fig. 5.**
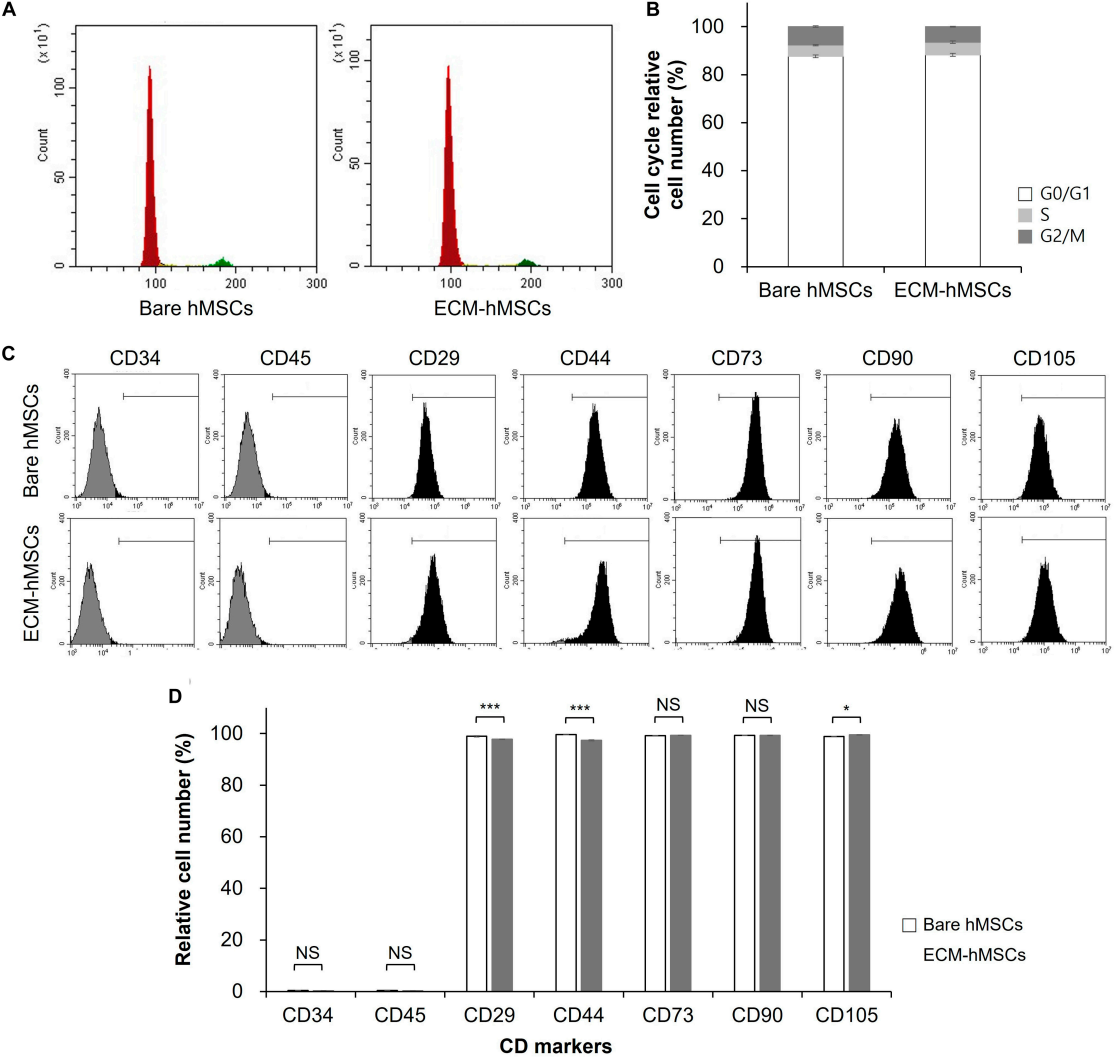
The cell cycle and maintenance of stemness of bare hMSCs and ECM-hMSCs. (A) Cell cycle analysis was quantified by PI-stained DNA of bare hMSCs and ECM-hMSCs on day 7 of cultures (*n* = 4). (B) The graph presented related cell number (%) of cell cycle on bare hMSCs and ECM-hMSCs from (A). (C) Stemness analysis to confirm stem cell characteristics by flow cytometry analysis. (D) The graph presented populations of hMSCs for stemness markers based on stemness analysis from (C) (*n* = 4, *P < 0.05 and ***P < 0.001).

We next assessed the effects of ECM coating on the cell cycle of hMSCs by evaluating the distribution of bare hMSCs and ECM-hMSCs in different stages of the cell cycle after 7 d in culture, using FACS analysis to quantify PI-stained DNA (Fig. 5A and B). This analysis showed that the percentages of bare hMSCs distributed to the G0/G1, S, and G2/M phase were 87.7% ± 0.6%, 4.7% ± 0.3%, and 7.7% ± 0.3%, respectively, values not significantly different from those of ECM-hMSCs, which showed a corresponding distribution of 88.3% ± 0.6%, 5.3% ± 0.5%, and 6.5% ± 0.2%. We next evaluated the stemness of bare hMSCs and ECM-hMSCs after 7 days in culture using a FACS analysis of positive and negative MSC stemness markers, based on ISCT guidelines [37]. The analysis (Fig. 5C) and subsequent quantitation (Fig. 5D) showed that similarly high percentages of cell populations in bare hMSC and ECM-hMSC groups (respectively) expressed the positive markers, CD29 (98.94% ± 0.25% and 97.81% ± 0.13%), CD44 (99.61% ± 0.04% and 97.36% ± 0.17%), CD73 (99.19% ± 0.07% and 99.30% ± 0.05%), CD90 (99.29% ± 0.11% and 99.28% ± 0.11%), and CD105 (98.89% ± 0.05% and 99.44% ± 0.10%). Bare hMSC and ECM-hMSC populations also expressed similarly low percentages of the negative markers CD34 (0.44% ± 0.06% and 0.35% ± 0.09% for bare hMSCs and ECM-hMSCs, respectively) and CD45 (0.37% ± 0.05% and 0.35% ± 0.03%, respectively).

### Cell-protective effects of ECM coating on hMSCs under external stress

**Fig. 6.**
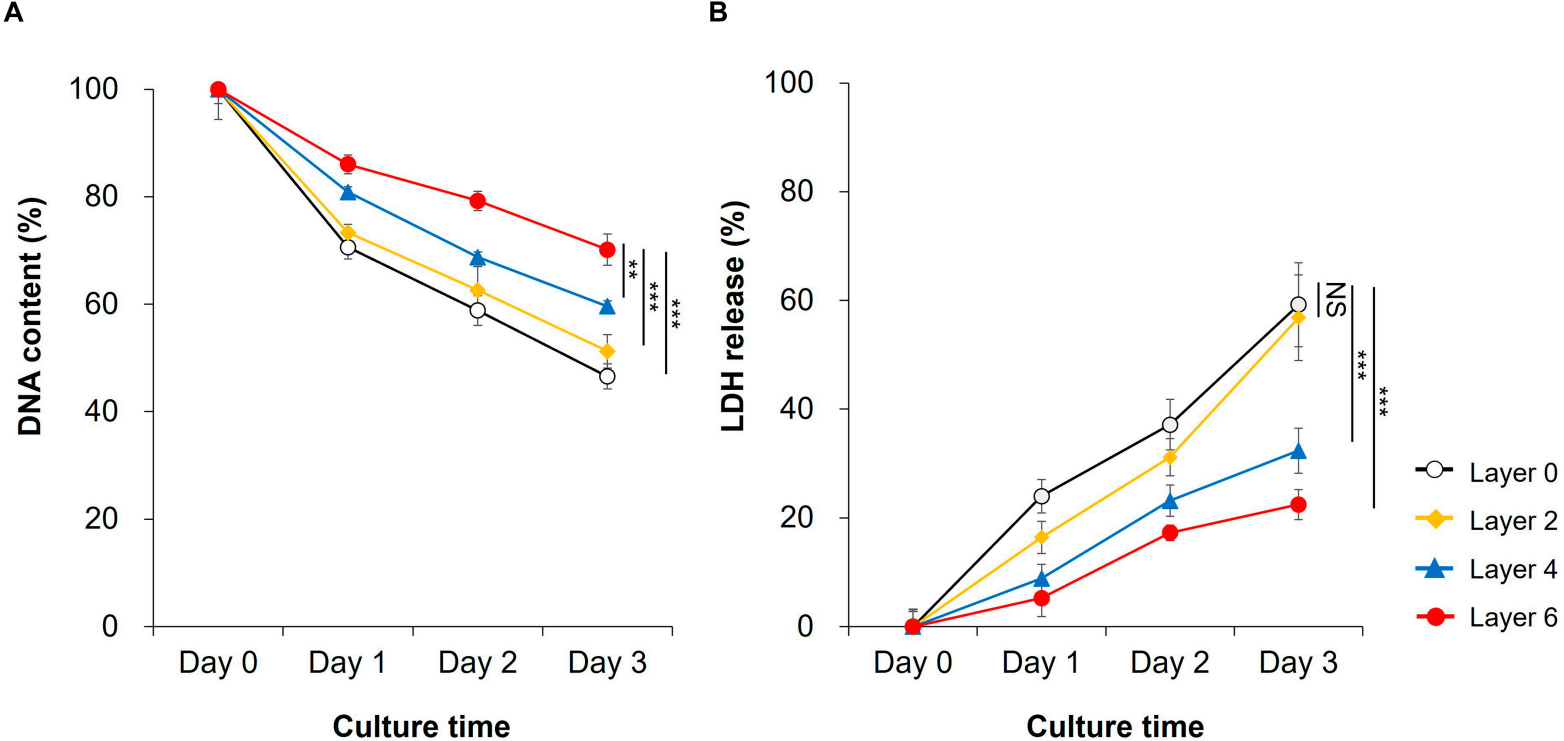
Cell-protective effect of ECM coating on hMSCs by coating layers under stress paradigms: low-attachment culture conditions (symbols indicate different ECM coating layers: white circle [Layer 0], yellow diamond [Layer 2], blue triangle [Layer 4], and red circle [Layer 6]). (A) DNA contents of both bare hMSCs and ECM-hMSCs under low-attachment conditions were detected using DNA assay (*n* = 4, **P < 0.01 and ***P < 0.001 at 3 days of culture). (B) Lactate dehydrogenase (LDH) release (%) of bare hMSCs and ECM-coated hMSCs (*n* = 4, ***P < 0.001 at 3 days of culture).

Finally, we evaluated the protective effects of ECM coating on hMSCs under 2 different stress paradigms: low-attachment growth conditions and exposure to a mechanical force. Under low-attachment conditions, cell viability tended to decrease over culture time in both groups (Fig. 6A). However, bare hMSCs and ECM-hMSCs were differentially affected by growth on low-attachment culture dishes. For bare hMSCs under these conditions, survival rates were 70.6% ± 2.2%, 58.8% ± 2.8%, and 46.6% ± 2.4% on culture days 1, 2, and 3, respectively. In contrast, under the same conditions, the survival rates for ECM-hMSCs on culture days 1, 2, and 3 were 86.0% ± 1.7%, 79.2% ± 1.8%, and 70.1% ± 2.9%, respectively. These values translate to corresponding increases of 21.8%, 34.6%, and 50.6% compared with those for bare hMSCs. The extent to which low-attachment conditions caused cell damage was evaluated by measuring LDH release from hMSCs. As shown in Fig. 6B, values of LDH release for bare hMSCs under low-attachment culture conditions were 24.0% ± 3.1%, 37.2% ± 4.6%, and 59.2% ± 7.7% on days 1, 2, and 3 of culture, respectively. For ECM-hMSCs (Layer 6) under the same conditions, LDH release values on days 1, 2, and 3 of culture were decreased to 5.2% ± 3.4%, 17.3% ± 1.5%, and 22.5% ± 2.8%, respectively. Compared with bare hMSCs, the percentage of damaged ECM-hMSCs was reduced by 78.2%, 53.6%, and 62.1% on days 1, 2 and 3, respectively. We also examined LDH release in 2-layer and 4-layer ECM-coated hMSCs under low-attachment conditions. We found that LDH release values for 2-layer ECM-coated hMSCs incubated for 1, 2, and 3 days were 16.4% ± 3.0%, 31.2% ± 3.4%, and 56.8% ± 7.9%, respectively, values that were somewhat lower than those for bare hMSCs. The relative release values for LDH on days 1, 2, and 3 in culture under these conditions were further decreased to 8.8% ± 2.6%, 23.2% ± 2.9%, and 32.4% ± 4.2% for 4-layer ECM-coated hMSCs. These results demonstrate the greater viability of ECM-coated hMSCs compared with bare hMSCs under low-attachment culture conditions.

**Fig. 7.**
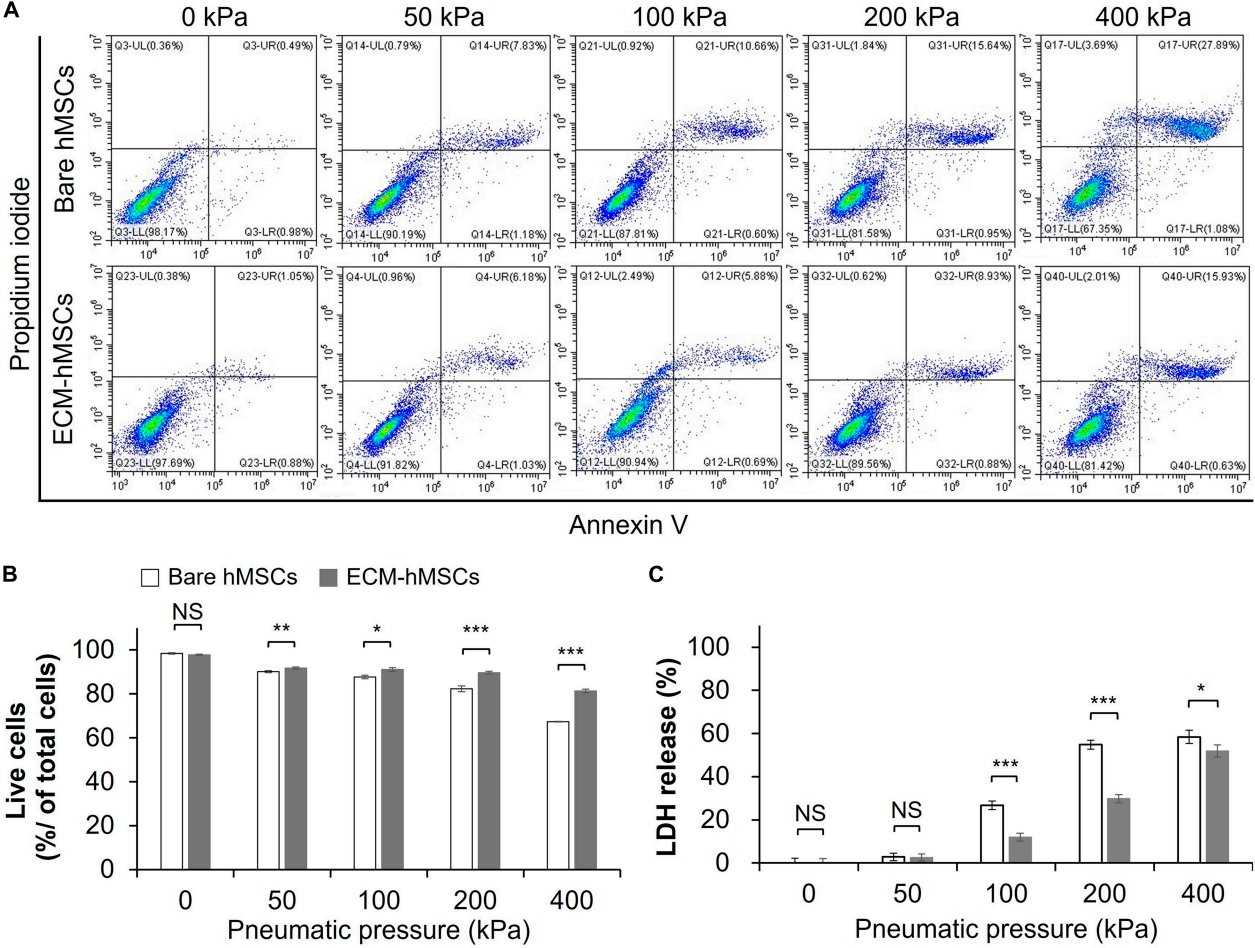
Cell-protective effect of ECM coating on hMSCs under stress paradigms: mechanical force. (A) The annexin V/PI images reveal the cytoprotective effect of ECM coating under mechanical force (*n* = 4). (B) The graph presented the percentage of live cells from (A) (*n* = 4, *P < 0.05, **P < 0.01, and ***P < 0.001). (C) LDH release (%) of hMSCs and ECM-hMSCs under mechanical force (*n* = 4, *P < 0.05, and ***P < 0.001).

Finally, we evaluated the protective effect of ECM coating on ECM-hMSCs against a mechanical force. To this end, we extruded hMSCs through a 21G needle under various pneumatic pressures to induce cell damage and evaluated the viability of ECM-hMSCs by measuring the ratio of annexin V-stained to PI-stained cells by FACS (Fig. 7). The quantitative results, calculated from Fig. 7A, are summarized graphically in Fig. 7B. The survival rate of bare hMSCs was inversely related to pneumatic pressure, decreasing from 98.4% ± 0.3% in controls (0 kPa), to 90.1% ± 0.5% at 50 kPa, 87.7% ± 0.8% at 100 kPa, 82.3% ± 1.3% at 200 kPa, and 67.4% ± 0.2% at 400 kPa. ECM-hMSCs showed smaller decreases in viability compared with 0 kPa controls (97.8% ± 0.3%) at 50 kPa (91.7% ± 0.5%), 100 kPa (91.0% ± 0.9%), 200 kPa (89.7% ± 0.6%), and 400 kPa (81.4% ± 0.8%). Thus, the ECM coating reduced the proportion of dead cells by 16.4%, 27.2%, 41.8%, and 43.0% at pneumatic pressures of 50, 100, 200, and 400 kPa, respectively, underscoring the significant cell-protective effect of this coating against pneumatic pressure. We further evaluated the extent of cell membrane damage and death attributable to different pneumatic pressures by measuring LDH release (Fig. 7C). This analysis showed that values for LDH release by bare hMSCs were 2.8% ± 1.8%, 26.7% ± 2.0%, 54.8% ± 2.1%, and 58.4% ± 3.1% at pneumatic pressures of 50, 100, 200, and 400 kPa, respectively. By comparison, values for LDH release by ECM-hMSCs under pneumatic pressures of 50, 100, 200, and 400 kPa were 2.5% ± 1.7%, 12.0% ± 1.9%, 29.8% ± 1.9%, and 51.9% ± 2.8%, respectively. The amount LDH released by hMSCs was similar at a pressure of 50 kPa, regardless of ECM coating, but decreased by 54.9%, 45.6%, and 11.2% at pneumatic pressures of 100, 200, and 400 kPa, respectively, compared with that of bare hMSCs. Collectively, these results underscore the highly effective cell-protective property of ECM coatings on hMSCs against the damaging effects of pneumatic pressure.

## Discussion

In this study, we fabricated ECM-coated hMSCs using the LbL technique as a strategy for protecting hMSCs and maintaining their cellular functions in the face of external stresses. We demonstrated that ECM coatings applied with the LbL technique stabilize cells and protect them from a variety of external stresses without interfering with cell function. These analyses determined that a 6-layer ECM coating offered the highest cell-protective effect, acting as the most stable and effective coating.

Cell membranes are negatively charged owing to the presence of outward-facing phosphates in the lipid bilayer [23,26]. Charge on the cell membrane can influence intracellular pathway regulation, cell structure and function, and cell development through changes in membrane permeability [38]. Changes in the charge of cell membranes can also cause various adverse effects, including imbalances in ion concentrations, disruptions in substance transport, and impairments in signal transduction and carcinogenesis, among others [38,39]. Because HyA is a negatively charged polysaccharide owing to the carboxyl group [34], we chose it as the final outer coating layer to mimic the negatively charged properties of the cell membrane.

ECMs coated on the surface of hMSCs were identified by fluorescence imaging using FACS and CLSM, which showed that the MFI of the ECM coating layer increased with deposition of increasing numbers of layers. MFI values increased significantly with a single coating of gelatin (Layer 1) and a further coating with HyA (Layer 2), and then gradually increased with additional coating layers. Some studies reported that the significant increase in fluorescence accompanying the first gelatin and HyA layers is attributable to hydrophobic interactions of PBS during the coating process [40]. The similar index of MFI between Layer 6 and Layer 8 indicated that HyA was already sufficiently coated around the cells by 6-layer ECM coating with 5-min coating per layer.

ECM coating did not change the average diameter of hMSCs, but did cause changes in the surface of the coated cell. These changes reflect nano-level coating of the micro-sized cell surface by the LbL method, consistent with previous reports on PC cells and retinal pigment epithelial cells [41,42]. The difference in morphology of the cell surface is due to the fact that the final HyA layer coat on the surface of hMSCs may aggregate because of the presence of EtOH during SEM image processing. The resulting granularity of the ECM-hMSC surface is in good agreement with previous reports on HyA [43].

The viability of ECM-coated hMSCs did not decrease significantly with increasing numbers of coatings up to Layer 8, as shown by FACS (Fig. 3A). Images of live/dead-stained ECM-coated hMSCs (Fig. 3C) showed that viability was minimally impaired by ECM coating that it was difficult to distinguish dead ECM-hMSCs after 3 days in culture. All ECM-coated hMSCs, regardless of the number of ECM layers, grew stably over time in culture. The proliferation rate of ECM-coated hMSCs after 1 and 3 days of culture decreased with each additional coating layer; interestingly, however, after 7 days in culture, the proliferation rates of 6-layer and 8-layer ECM-coated hMSCs increased by more than 29.4%, and 44.0%, respectively, compared with that of bare hMSCs. The initial decrease is presumably attributable to the lack of initial adhesion between the cell and substrate, which delays cell spreading and development [44]. However, the increase in growth rate in 6-layer and 8-layer ECM-coated hMSCs is thought to be caused by the various growth factors secreted by cells during the 7-day incubation period, which accumulate at high concentrations on hMSCs by electrostatically combining with the rich coating in Layers 6 and 8. As the growth factor-binding coating layer degrades, the bound growth factors are slowly released, abetting cell growth. Gelatin and HyA used in the LbL-applied coating might also directly enhance proliferation and migration of hMSCs during the culture period [45,46].

Based on the above results, we performed a follow-up study of 6-layered hMSCs (ECM-hMSCs) to evaluate cell cycle and stem cell characteristics. Important physiological processes, together with morphological stability, must be maintained. We found that the distribution of ECM-hMSCs in different cell cycle stages was not significantly different from that of bare hMSCs, and there was no evidence that the ECM coating caused cell damage or stress. A subsequent evaluation of stem cell markers for MSCs showed that, after ECM coating, the population of ECM-hMSCs expressing positive stemness markers (CD29, CD44, CD73, CD90, and CD10) was greater than 97.3%, whereas the population expressing negative stemness markers (CD34 and CD45) was less than 0.5%. These findings align with ISCT guidelines for defining MSC stem cells, which proposed criteria of greater than 95% positivity for the indicated positive stemness markers and less than 2% positivity for negative stemness markers [37]. Collectively, these observations confirm that there was no significant change in the unique biological characteristics of hMSCs, including stemness maintenance and cell cycle, in ECM-hMSCs using the LbL technique.

Next, we assessed the efficacy of the LbL-mediated ECM coating in resisting physical external stress by determining the viability of ECM-hMSCs exposed to 2 different stresses: culture on a low-attachment substate and mechanical shear force. It is well known that anchorage-dependent cells such as MSCs require cell–cell interactions as well as cell–ECM interactions to survive [47]. However, it is also the case that a single cell isolated from a culture dish for injection may be exposed to compressive forces at the application site and to shear stress in an injection needle without interacting with other cells or ECM [14,17]. In both cases, isolated hMSCs could be killed, either as a result of anoikis caused by the loss of cell–substrate contact or because of the damage to cell membranes induced by shear stress [14]. We found that hMSCs cultured on low-attachment culture plates (to prevent cell–ECM interactions) showed a significant initial (after 1 day in culture) increase in viability with increasing numbers of coatings. After 3 days in culture, viability, measured as DNA content (%), increased by 10%, 28%, and 50.6% in 2-layer, 4-layer, and 6-layer hMSC, respectively, compared with bare MSCs. As culture time increased, the rate of DNA reduction varied for hMSCs with different numbers of ECM layers, with 6-layer hMSCs exhibiting the smallest reduction. This finding contrasts with MTT assay results shown in Fig. 3D. Under normal hMSC culture conditions, the growth rate of 6-layer hMSCs (i.e., ECM-hMSCs) was less than that of bare hMSCs over 3 days of culture, but this was reversed after 7 days of culture, when the growth rate of ECM-hMSCs was greater than that of bare hMSCs, reflecting the protective action of the ECM coating of ECM-hMSCs. In contrast, the initial growth (i.e., after 1 day of culture) of ECM-hMSCs cultured under low-attachment conditions was more than 21.8% higher than that of bare hMSCs, as shown in Fig. 6. The initial increase in viability was attributed to the fact that the RGD of coated gelatin and α5β1 integrin receptor on the surface of ECM-hMSCs was already bound to each other prior to incubation, despite incubation under low-attachment conditions. These results are consistent with previous reports in which anoikis was blocked using synthetic molecules, proteins, or growth factors to promote binding of isolated cells to α5β1 integrin and increase cell adhesion [48]. They also demonstrate that ECM coated onto hMSCs can influence cell–ECM interactions essential for initial growth in culture.

As an additional indicator of cellular damage, we also monitored the LDH-release behavior of hMSCs cultured under low-attachment conditions. Since LDH is released from damaged cell membranes, any reduction in LDH secretion by ECM coating would indicate reduced cell membrane damage and thus a protective effect. Consistent with our DNA assay results, LDH release from ECM-coated hMSCs under low-attachment conditions also decreased with increasing number of coating layers after 3 days in culture. The initial LDH secretion from 6-layer ECM-coated hMSCs (ECM-hMSCs) decreased by 78.2% compared to that of bare MSCs after 1 day in culture. Collectively, our results show that coating with an ECM consisting of gelatin and HyA could aid the proliferation of hMSCs while suppressing the apoptotic hMSC death that may occur under external stress conditions, such as culture on low-attachment substrates.

Continuing our investigation of cell-protective effects of ECM coating against physical external stresses, we compared survival rates of bare hMSCs and ECM-hMSCs subjected to mechanical shear stress induced by pneumatic pressure, using cell viability and LDH release as indicators of cell damage. Although the percentage of injured or dead cells increased with increasing pneumatic pressure for both bare hMSCs and ECM-hMSCs (Fig. 7), there were significantly fewer such cells among the ECM-hMSC population, indicating better resistance to pneumatic pressure compared with bare hMSCs. We also found that, although hMSCs showed similar LDH-release values regardless of ECM coatings at pressure extremes (≤50 kPa or ≥400 kPa), the survival rate of ECM-hMSCs exposed to pneumatic pressures of 100 or 200 kPa improved by 54.9% and 45.6%, respectively, compared with that of bare hMSCs. These results show that the survival rate of hMSCs subjected to pneumatic pressures below 200 kPa can be maintained above 91% by ECM coating alone.

It is well known that the ECM provides mechanical support for cells, facilitates cell movement, and mediates cell–cell interactions [2,27]. The ECM components, gelatin and HyA, in the LbL coating on hMSCs bind to α5β1 integrin and CD44, respectively. This supposition is in good agreement with previous studies on the biological interactions of the RGD sequence in gelatin and CD44 in HyA on the surface of cells showing that blocking α5β1 integrin and CD44 binding sites decreases the amount of gelatin and HyA attached to cells [40,48]. HyA absorbs water and serves as a lubricant in tissues such as skin, synovial fluid, and cartilage [32]. Viscoelasticity conferred by its high water content and molecular flexibility enables it to act as a shock absorber [33]. HyA could protect cells and maintain structural stability under mechanical stresses such as shear force or pressure during injection. Our findings indicate that it is not only the biological properties of gelatin and HyA that matter for the protective effects of ECM, but also cell-bound gelatin and HyA, and the rigid electrostatic binding between gelatin and HyA, which can blunt the physical forces imparted on bare hMSCs. Thus, we conclude that ECM coating using the LbL technique improves the resistance of ECM-hMSCs against external stresses, such as low-attachment conditions and mechanical force.

In conclusion, to minimize adverse effects on cells caused by external stress and create a safe cell coating film that does not affect cell characteristics, we introduced a thin protective film on the cell surface composed of a cell-friendly ECM. ECM-coated hMSCs were successfully prepared using LbL technology without altering the intrinsic biological characteristics of these stem cells. The ECM coating acted as a barrier that safely protected cells from external stress without affecting cell viability or physiological function. These studies thus establish a coating method capable of resisting various external stresses that may occur during cell culture and cell therapy applications—a method that could be expanded to various other applications in the future. However, some limitations remain, especially given that biological systems are exposed to a variety of physical stresses and biological responses. Among potential stresses, we showed that interfering with contact between the cell and substrate or application of direct physical force to the cell during cell culture greatly affects cell survival and physiological activity [6,49,50]. Such external stress can arise not only during cell injections for cell therapy, but also in nozzle-based applications used in clinic and research settings, including 3D-bioprinting and microfluidic systems. This study presents a new strategy for enhancing cell survival in vivo, improving the efficacy of cell therapy, and expanding the scope of application tissue engineering technology. Furthermore, preclinical trials are currently evaluating the therapeutic efficacy of coating cells on musculoskeletal models.

## Data Availability

All data generated or analyzed in this study are available within the article and the Supplementary Materials. Additional information can be obtained from the corresponding author upon reasonable request.
