# Supplementary material for: External-Force-Offset Effects of ECM Coating Layers on hMSCs Subjected to External Physical Force
Source: Biomater Res. 2025 Oct 3;29:0265. doi: 10.34133/bmr.0265 (PMC12491781; doi:10.34133/bmr.0265)
Supplement: Supplementary 1 — Fig. S1 [file bmr.0265.f1.docx]

**Supplementary Materials**


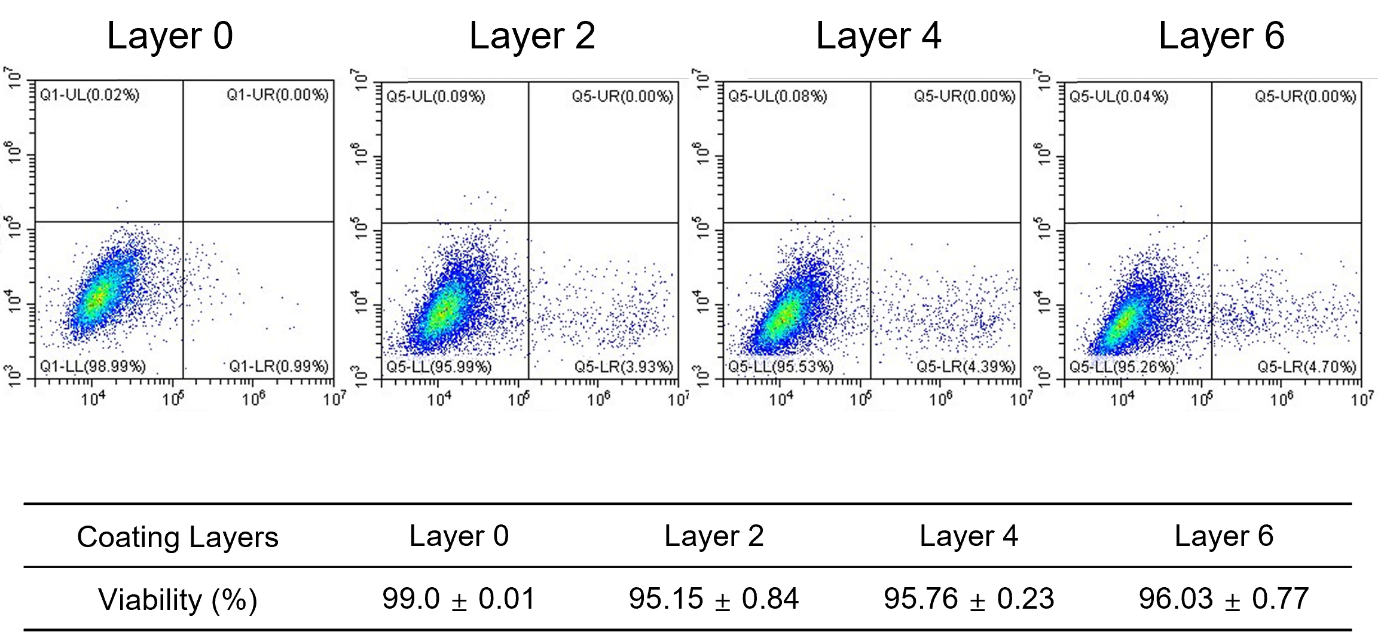


**Fig. S1** Viability analysis of bare hMSCs and ECM-hMSCs after 10 min-coating per layer. FACS analysis using Annexin V/PI staining showed the cell distribution at 10 min-coating per layer (top). A summary table presents the percentage of cell viability at 10 min-coating per layer based on FACS analysis (bottom). (n=4)
